# Structural‐Functional Dissociation in TBI Hemiplegia: Meridian‐Sinew Therapy Promotes Motor Recovery Despite Corticospinal Tract Damage—A Case Report

**DOI:** 10.1002/ccr3.71296

**Published:** 2025-10-21

**Authors:** Juyue Hong, Yuchun Zheng, Gengbiao Zhang, Hongyi Zheng, Jinghua Wu, Wenbin Zheng

**Affiliations:** ^1^ Department of Radiology The Second Affiliated Hospital, Shantou University Medical College Shantou Guangdong China; ^2^ Shantou Longhu District Jinrou Traditional Chinese Medicine Research Institute Shantou China; ^3^ Beijing Jinrou Traditional Chinese Medicine Research Institute Beijing China

**Keywords:** diffusion tensor imaging, hemiplegia, magnetic resonance imaging, meridian‐sinew therapy, neuroplasticity, rehabilitation, traditional Chinese medicine, traumatic brain injury

## Abstract

This study presents neuroimaging analysis of a 31‐year‐old male patient with hemiplegia secondary to severe traumatic brain injury who was treated with an external therapy of Traditional Chinese Medicine (TCM) called meridian‐sinew therapy. The patient exhibited complete right‐sided limb paralysis following trauma, with full muscle strength recovery after therapy. Serial diffusion tensor imaging evaluations revealed persistently reduced fractional anisotropy values in the left corticospinal tract (CST.L) below the normal range posttreatment, indicating a structure–function decoupling phenomenon. Despite incomplete restoration of structural integrity in CST.L, significant motor function improvement was observed. The findings suggest that meridian‐sinew therapy may promote motor recovery through activation of compensatory networks involving non‐CST pathways. This investigation provides neuroimaging evidence supporting the therapeutic efficacy of meridian‐sinew therapy in TBI‐induced hemiplegia and offers novel insights into potential mechanisms of action.

**Summary**

- Meridian‐sinew therapy may enable motor recovery in TBI‐induced hemiplegia through non‐CST compensatory pathways, despite persisting corticospinal tract damage.
- Neuroimaging supports its role in neural network reorganization.

## 1 Introduction

Traumatic brain injury (TBI) is one of the leading causes of death and disability worldwide [1] and places a significant burden on healthcare systems [2]. Patients with TBI frequently experience motor dysfunction issues, including hemiplegia [3], and early rehabilitation intervention is crucial for patients' recovery [4, 5]. Traditional Chinese Medicine (TCM) physiotherapies, such as acupuncture and tuina, demonstrate unique advantages in improving motor function following TBI through their multi‐target regulatory mechanisms [6, 7, 8, 9]. Moreover, the regulation of neurotransmitter release and facilitation of neural regeneration have been identified as one of the pivotal underlying mechanisms by which acupuncture ameliorates TBI‐associated deficits [10, 11]. Meridian‐sinew therapy, a critical branch of TCM physiotherapy, employs mechanical stimulation modalities (e.g., tuina, acupuncture) to stimulate fascial tissues and regulate qi‐blood circulation, demonstrating clinically significant efficacy in ameliorating motor dysfunction and facilitating comprehensive rehabilitation in hemiplegic patients. Nevertheless, its therapeutic mechanisms and outcomes remain underexplored using neuroimaging evidence. Diffusion tensor imaging (DTI) provides a sensitive modality for detecting alterations in the microstructural integrity of white matter tracts through quantitative assessment of fractional anisotropy (FA) values. We present the case of a 31‐year‐old male TBI patient with hemiplegia demonstrating FA alterations in white matter tracts through pre‐ and post‐meridian‐sinew therapy DTI analysis. This investigation provides empirical neuroimaging evidence supporting the therapeutic efficacy of meridian‐sinew therapy in TBI‐related hemiplegia, and establishes novel conceptual paradigms for investigating the underlying therapeutic mechanisms.

## 2 Case Presentation

### 2.1 Medical History

**FIGURE 1:**
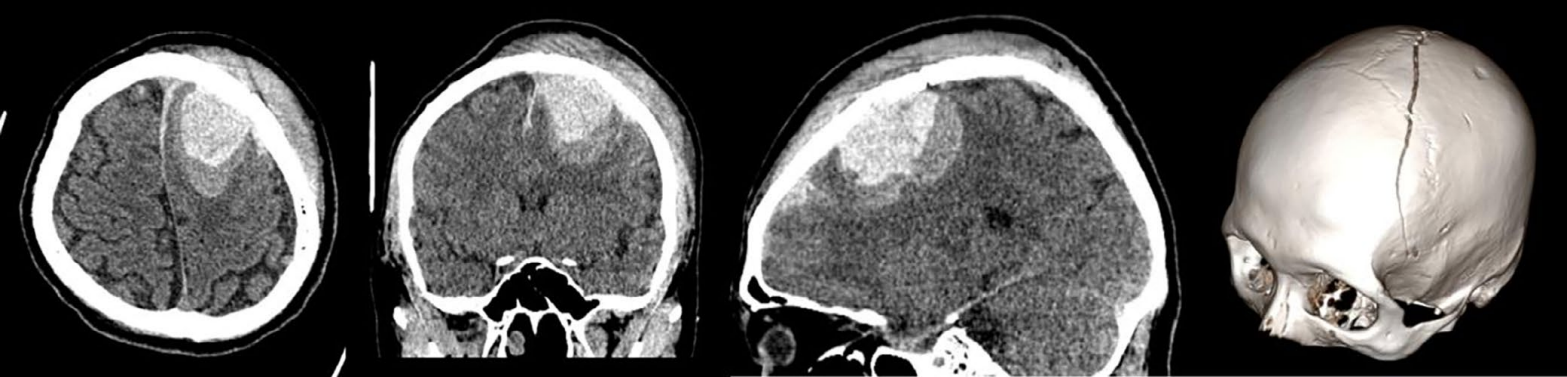
Computed tomography (CT) images showing skull fracture, intracerebral hematoma, subdural hemorrhage as well as multiple punctate hemorrhagic foci caused by TBI.

A 31‐year‐old right‐handed male sustained a TBI following a headfirst impact during an electric bicycle accident. Immediate post‐traumatic manifestations included loss of consciousness and unresponsiveness to verbal stimuli. Upon hospital admission, the patient presented with right‐sided hemiplegia and recurrent projectile vomiting of gastric contents. Neurological examination revealed altered mental status characterized by eye opening in response to verbal commands but without meaningful verbal response, right‐sided flaccid paralysis (Medical Research Council [MRC] Grade 0), preserved left‐sided motor strength (MRC Grade IV), and a Glasgow Coma Scale (GCS) score of 10. Initial cranial computed tomography (CT) revealed a large left frontal intracerebral hematoma (57 × 36 mm in axial dimensions) with concomitant left frontal subdural hemorrhage. Additional imaging findings included multiple punctate hemorrhagic foci in the right frontal and bilateral parietal lobes, radiologically suggestive of diffuse axonal injury (Figure 1). The patient underwent an emergent left frontal craniotomy for hematoma evacuation. Postoperative neurological evaluations performed 1 month after the injury demonstrated persistent right‐sided flaccid paralysis (MRC Grade 0) with preserved left‐sided motor strength (MRC Grade V). Despite adherence to a 15‐day standardized neurorehabilitation protocol at a tertiary rehabilitation center following discharge, no significant functional improvement in right hemiplegia was documented.

### 2.2 Therapeutic Intervention

Following informed consent for meridian‐sinew therapy participation and neuroimaging investigations, the patient initiated a structured meridian‐sinew intervention protocol 1 month after the injury. The therapeutic protocol adopted “Wu Jinghua's Jingjin (meridian‐sinew) Therapy” [12, 13], a holistic regimen centered on untying the sinew nodules and dredging the qi of the meridians, along with a variety of professional massage techniques and acupuncture treatment. This therapeutic approach modulates the condition of the meridian‐sinew system, including muscles, tendons, ligaments, and other soft tissues, to achieve the goals of pain relief and functional restoration. The “Fascia Region Needling Method (Jing Jin Ci Fa)” was employed for acupuncture administration: needles were left in place for roughly 1 h each time, with the primary focus on untying the detected sinew nodules and fascia exhibiting abnormalities. Simultaneously, specific tuina guided by meridian‐sinew theory was performed to loosen the sinew nodules throughout the body for 40 min. This intervention was administered once a day, for 4 consecutive days a week, with 3 days off.

### 2.3 Multimodal Imaging Assessment

The patient underwent four MRI scans over a seven‐month period following the injury, spanning both pre‐therapeutic baseline and post‐therapeutic follow‐up phases. These scans included DTI, three‐dimensional T1‐weighted (3D‐T1) imaging, and were complemented by MRC scale assessments at multiple time points. DTI data were acquired using a single‐shot spin‐echo echo‐planar imaging (SS‐SE‐EPI) sequence with the following parameters: repetition time (TR) = 8000 ms; echo time (TE) = 99.3 ms; number of excitations (NEX) = 1; slice thickness = 4 mm; interslice gap = 0 mm; acquisition matrix = 128 × 128; field of view (FOV) = 240 × 240 mm; 15 diffusion‐encoding directions; minimum *b*‐value = 0 s/mm^2^; maximum *b*‐value = 1000 s/mm^2^. DTI data were processed using the PANDA software (https://www.nitrc.org/projects/panda/) to quantify white matter FA. After DICOM‐to‐NIfTI conversion and eddy current/motion correction, brain‐extracted FA maps were nonlinearly normalized to MNI‐152 space using diffeomorphic registration (DARTEL algorithm). Region‐specific FA values were computed across 48 white matter tracts defined by the JHU‐ICBM‐DTI‐81 probabilistic atlas, with voxelwise measurements restricted to spatially thresholded masks (probability > 60%, FA > 0.2). Following image acquisition, the DTI datasets were transferred to an offline workstation AW4.3 (GE Medical system, advantage window 4.3) for postprocessing. The Functool software was employed to measure the results of the DTI datasets. During the measurement process, spatial geometric distortion correction was first applied to the DTI images to partially eliminate the geometric distortions caused by the EPI sequence. Seed ROIs and Target ROIs were manually set to track and reconstruct the corticospinal tract.

**FIGURE 2:**
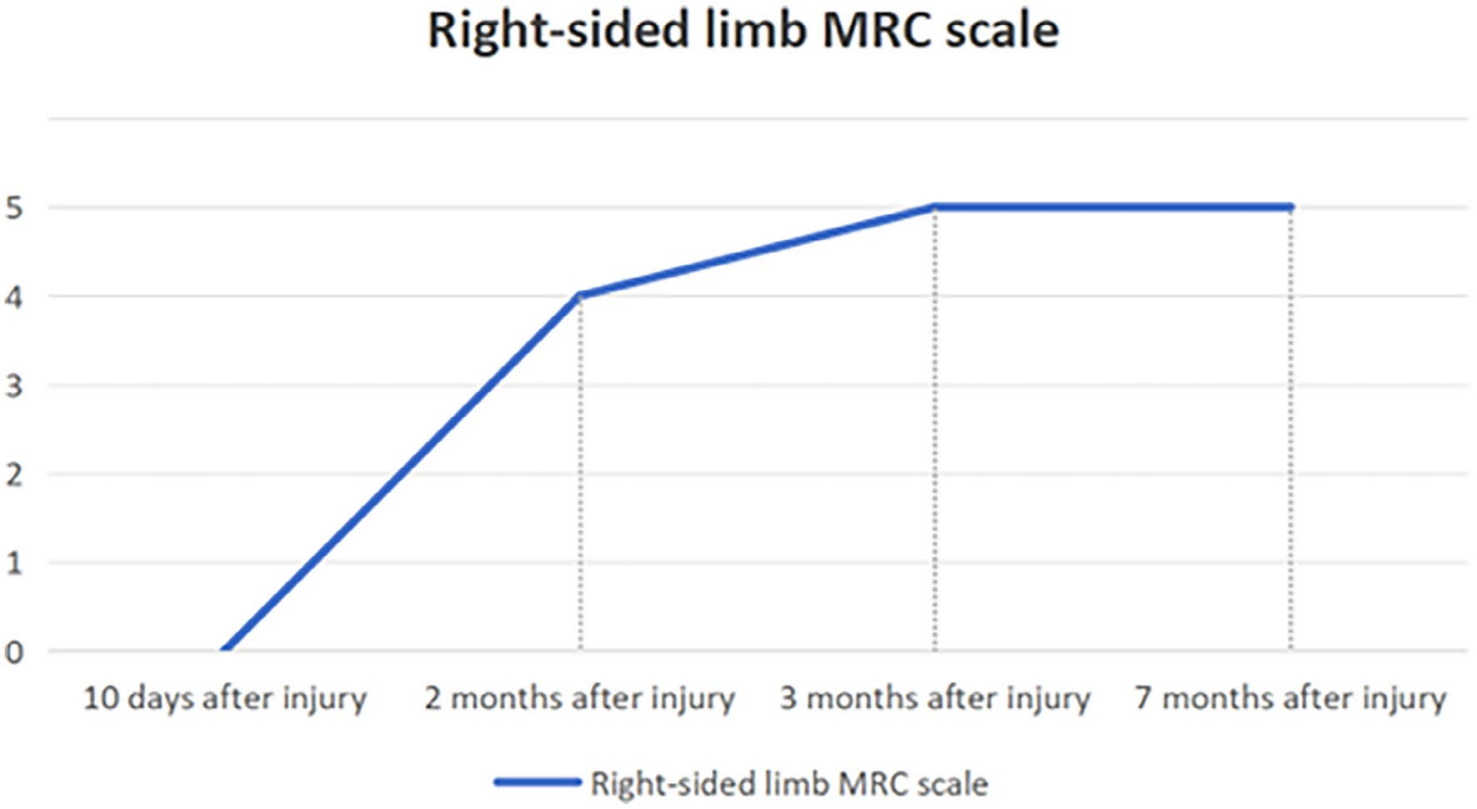
Trends in the Medical Research Council (MRC) scale score for the right limb before and after meridian‐sinew therapy. The horizontal coordinate is the date of the patient's imaging examination.

**FIGURE 3:**
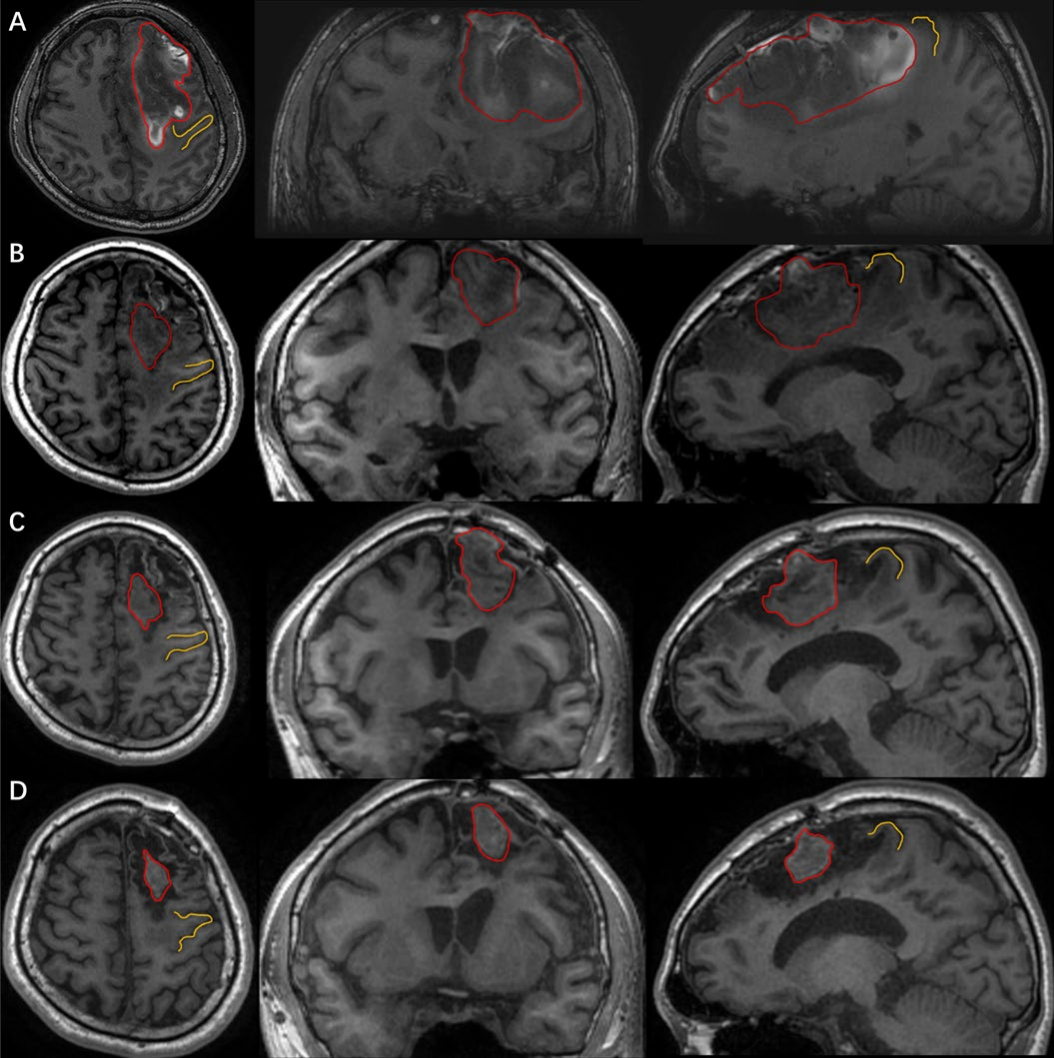
Three‐dimensional T1‐weighted imaging (3D‐T1WI) reveals volumetric and morphological changes of the intracranial hematoma and perihematomal region pre‐ versus post‐treatment: (A) 10 days after injury; (B) 2 months after injury; (C) 3 months after injury; (D) 7 months after injury, with marked hematoma absorption and alleviated compression on the adjacent precentral gyrus; red lines outline hematoma ranges at different stages, and yellow lines mark the precentral gyrus.

**FIGURE 4:**
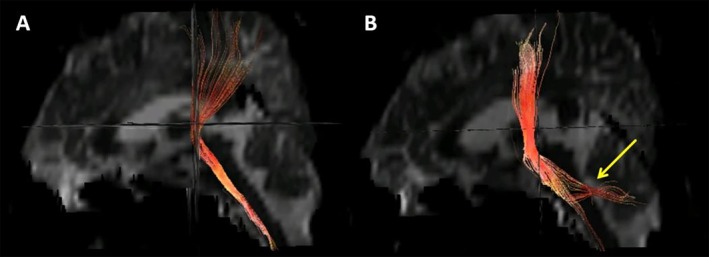
Sagittal diffusion tensor tractography (DTT) of the left corticospinal tract (CST.L, red fibers) displays pre‐treatment (A, 10 days post‐injury) and post‐treatment (B, 3 months post‐injury) findings, with aberrant bifurcations (yellow arrow) identified at the superior cerebellar peduncle level.

Following meridian‐sinew therapy, the patient exhibited progressive amelioration of right‐sided hemiparesis. MRC scale of the right limb has progressively improved and has now reached a level consistent with that of a healthy individual (Figure 2). On imaging, 3D‐T1 showed that the hematoma signals in the injured brain area were persistently reduced after intracranial surgery. The hematoma area was significantly absorbed and showed a gradual shrinking trend, and the compression status of the precentral gyrus adjacent to the hematoma was significantly improved compared with the previous state (Figure 3). We compared the measured FA values of the patients before and after treatment with 15 age‐matched healthy male controls (mean age 29.27 years, range 24–46 years) (Table 1). As in previous studies, FA values greater than or less than two standard deviations (SDs) compared with the control group were defined as abnormal [14]. Compared with control subjects, the left corticospinal tract and corpus callosum FA values showed no abnormalities before the untreated treatment but decreased significantly after the treatment. Mid cerebellar peduncle FA values showed abnormally lowered before untreated but continued to increase after treatment and recovered to be indistinguishable from normal controls. The superior cerebellar peduncle (SCP) showed abnormally increased values before untreated but returned to normal values after treatment. And diffusion tensor tractography (DTT) reconstructed after treatment (at 3 months post‐injury) demonstrated aberrant bifurcations originating from the left corticospinal tract (CST.L, red fibers) at the SCP level (Figure 4). The left medial lemniscus showed abnormally decreased FA values before untreated but increased to normal values after treatment. In addition, the FA values of the left posterior limb of the internal capsule were abnormally low but evolved in a “V‐shape” before and after treatment, showing a first decrease and then an increase. The results of the fourth examination (7 months post‐injury) showed that, except for the FA values of the CST.L and the body of the corpus callosum (BCC), which remained significantly lower compared with those of the normal control group, there were no differences in FA values in other regions (Table 1).

**TABLE 1:** Fractional anisotropy values in key abnormal white matter fiber tracts: Comparison between patients and healthy controls.

FA	Ten days after injury		Two months after injury		Three months after injury		Seven months after injury		Controls	
	Left	Right	Left	Right	Left	Right	Left	Right	Left	Right
CST	0.444	0.463	0.372**	0.442	0.375**	0.443	0.391**	0.454	0.459 ± 0.028	0.449 ± 0.027
SCP	0.483**	0.469**	0.398	0.398	0.400	0.391	0.396	0.392	0.419 ± 0.024	0.415 ± 0.019
ML	0.412**	0.478	0.476	0.487	0.482	0.490	0.485	0.486	0.505 ± 0.034	0.512 ± 0.031
MCP	0.263**		0.349		0.351		0.346		0.386 ± 0.022	
BCC	0.460		0.338**		0.330**		0.317**		0.452 ± 0.033	
PLIC	0.516**	0.594	0.475**	0.598	0.502**	0.583	0.516**	0.578	0.600 ± 0.029	0.618 ± 0.020

*Note:* FA values in the control group are presented as mean ± SD.Values marked with “**” exceed ±2 standard deviations from the mean of the control group.

Abbreviations: BCC, body of the corpus callosum; CST, corticospinal tract; FA, fractional anisotropy; MCP, middle cerebellar peduncle; ML, medial lemniscus; PLIC, posterior limb of the internal capsule; SCP, superior cerebellar peduncle.

## 3 Discussion

This case report documents a characteristic “structure–function decoupling” phenomenon in motor compensation following meridian‐sinew therapy for severe TBI. Specifically, a 31‐year‐old male patient achieved complete recovery of muscle strength (MRC Grade 5) in his right hemiplegic limb (initial MRC Grade 0) after meridian‐sinew therapy, despite persistently reduced FA values in the CST.L that remained below normal ranges during follow‐up assessments. This finding challenges the conventional paradigm that CST white matter integrity restoration is decisive for motor recovery and prognosis, thereby suggesting that meridian‐sinew therapy may facilitate functional recovery through alternative mechanisms, necessitating multidimensional analysis of neural network compensation.

To further unpack the biological basis of these alternative mechanisms, we first analyzed the temporal evolution of CST.L FA values. Notably, CST.L FA showed no significant decline in the early postoperative phase but exhibited progressive reduction during the period of muscle strength recovery post‐therapy—a pattern that aligns with the pathological progression of Wallerian degeneration (where distal axonal degeneration induces myelin breakdown and reduced water diffusion anisotropy). Intriguingly, the observed dissociation between progressive motor recovery and CST.L structural deterioration implies compensatory activation of non‐CST pathways. This speculation is supported by findings from Sinke et al. [15], who demonstrated in murine models that the reticulospinal and vestibulospinal tracts can compensate for CST damage by enhancing the synaptic plasticity of spinal interneurons. In this case, meridian‐sinew therapy may activate analogous compensatory networks via two potential mechanisms. First, mechanical stimulation from tuina activates Aδ fibers in the spinal dorsal horn. These fibers then directly excite reticulospinal motor neurons through propriospinal connections [16]. Second, acupuncture at Zusanli (ST36) enhances proprioceptive input, which is transmitted to the cerebellar cortex via the medial lemniscus, ultimately potentiating vestibulospinal regulation of axial musculature [17].

Beyond the compensatory activation of non‐CST pathways, FA changes in other key white matter structures provide additional insights into the patient's recovery. For instance, concurrent FA reduction in the corpus callosum body (paralleling CST.L trends) may reflect impaired interhemispheric integration. Despite this, the patient's recovery likely stemmed from dual compensatory mechanisms: (1) Enhanced contralateral (right) motor cortex output through preserved CST.R [18]; (2) Cortico‐bulbar tract modulation of reticulospinal pathways via spinal interneurons [16]. Complementary evidence also emerges from alterations in cerebellar‐brainstem circuits. The middle cerebellar peduncle (MCP) exhibited abnormally reduced FA, while the SCP showed elevated FA. Consistent with previous research [19], this pattern suggests compensatory hyperactivation of SCP through enhanced fiber alignment or alternative pathway recruitment to offset MCP dysfunction, which aligns with Morton's findings [17] on cerebellar nuclei‐mediated motor coordination. Post‐therapy elevation of MCP FA further supports this interpretation, as it is consistent with studies showing that acupuncture enhances cerebellar‐cortical connectivity [20]. Animal models also confirm that electroacupuncture improves cerebellar synaptic plasticity and motor recovery [21]. Although direct tractography was unavailable, the dynamic changes in MCP FA suggest axonal remodeling via collateral sprouting [22]. Notably, the subsequent normalization of SCP FA may indicate a transition from compensatory hyperactivation to homeostatic function. Furthermore, DTT reconstruction demonstrated aberrant bifurcations originating from the CST.L at the SCP level, with their terminal ends dispersed and terminating in the ipsilateral dentate nucleus (DN), cerebellar white matter, and surrounding vermis. These structural anomalies exhibited temporal correspondence with SCP FA dynamics (abnormally elevated pretreatment and restored posttreatment; Table 1), suggesting compensatory structural alterations in the cerebellar‐brainstem circuitry, which may underlie the potential neural reorganization for motor function recovery.

In addition to cerebellar‐brainstem circuit remodeling, dynamic changes in FA values of the left medial lemniscus reveal another critical neural mechanism by which meridian‐sinew therapy promotes motor recovery. At baseline, the left medial lemniscus exhibited significantly reduced FA; following meridian‐sinew therapy, however, FA markedly increased and ultimately normalized to within the reference range. Previous studies have demonstrated the presence of neural stem cells (NSCs) in the hypothalamus of mammals [23]. Acupuncture has been shown to promote neurogenesis and neural repair by inducing the proliferation and differentiation of endogenous NSCs [11, 24]. Further evidence suggests that acupuncture enhances the activity of glutamatergic neurons in the lateral hypothalamic area, which contributing to motor function recovery in patients with spinal cord injuries [25]. Additionally, proprioceptive afferent input is strongly associated with motor rehabilitation [26, 27]. Based on these findings, we hypothesize that meridian‐sinew therapy facilitates functional recovery through the following multimodal mechanisms: Mechanical stimulation from acupuncture and manual therapy directly activates proprioceptive receptors, transmitting sensory signals via the medial lemniscus to the primary somatosensory cortex. Enhanced neural input potentiates excitatory neurotransmitter release from lateral hypothalamic glutamatergic neurons through thalamocortical circuits. Chemotactic gradients of locally secreted chemokines guide the migration of newly generated NSCs to lesioned areas [28]. Collectively, these processes may drive axonal remodeling and synaptic plasticity within the damaged medial lemniscus, thereby enabling compensatory restoration of motor function.

Finally, the FA values of the left posterior limb of the internal capsule (PLIC.L) exhibited a “V‐shaped” trajectory over the treatment course—characterized by an initial decline followed by a subsequent recovery. This dynamic pattern first aligns with the pathological process of early Wallerian degeneration. Notably, the subsequent recovery phase of PLIC.L FA values further suggests structural remodeling of the cortico‐bulbar tract, a key pathway involved in motor signal transmission. Such remodeling is hypothesized to potentially strengthen the functional connections between the cortico‐bulbar tract and the vestibulospinal/reticulospinal pathways—alternative motor networks that have been implicated in compensatory motor recovery when the CST is damaged. Ultimately, this enhanced connectivity may facilitate more effective activation of spinal motor neurons, thereby contributing to the observed improvement in the patient's motor function despite persistent CST structural impairment.

This study still has several notable limitations. First, as a case report, the corresponding results may be subject to observational bias; therefore, it is necessary to validate these results through well‐designed cohort studies in the future. Second, the study lacks multimodal validation, such as collaborative verification using techniques like functional MRI and transcranial magnetic stimulation. In the future, integrating DTI with other technologies to improve multimodal imaging studies will help obtain more comprehensive data. Finally, optimizing individualized meridian‐sinew therapy protocols based on patient conditions to achieve optimal neurorehabilitation outcomes also represents a clinically significant area of research.

## 4 Conclusion

This report presents neuroimaging evidence confirming the efficacy of meridian‐sinew therapy in TBI‐related hemiplegia and provides novel insights into its underlying mechanisms. Three key scientific questions emerge from the findings: (1) the nonlinear relationship between white matter integrity metrics (e.g., FA) and functional outcomes; (2) the spatiotemporal activation patterns of multimodal compensatory networks; and (3) the competition or synergy between compensation mechanisms during critical time windows.

The findings carry two clinical implications. First, prognostic evaluation of TBI patients should transcend single‐tract structural analysis. Instead, it should incorporate multimodal functional and metabolic assessments. Second, rehabilitation strategies should target specific compensatory pathways. This is because meridian‐sinew therapy demonstrates multi‐mechanistic neuromodulatory potential that deserves further clinical exploration.

## Author Contributions

**Juyue Hong:** writing – original draft, writing – review and editing. **Yuchun Zheng:** formal analysis, investigation, writing – original draft, writing – review and editing. **Gengbiao Zhang:** software, Writing – review and editing. **Hongyi Zheng:** resources. **Jinghua Wu:** resources. **Wenbin Zheng:** conceptualization.

## Ethics Statement

The Ethics Committee of Second Affiliated Hospital of Shantou University Medical College approved the experimental protocol (No. 2022‐120), and all volunteers were informed of the precautions for MRI and signed the informed consent form.

## Consent

Written informed consent was obtained from the patient to publish this report in accordance with the journal's patient consent policy.

## Conflicts of Interest

The authors declare no conflicts of interest.

## Data Availability

The data supporting the findings of this study are not publicly available due to patient privacy concerns. However, the data can be made available from the corresponding author upon reasonable request and with permission from the relevant third party.
